# Pay for performance program reduces treatment needed diabetic retinopathy - a nationwide matched cohort study in Taiwan

**DOI:** 10.1186/s12913-018-3454-6

**Published:** 2018-08-15

**Authors:** Shwu-Jiuan Sheu, Wen-Liang Lin, Yea-Huei Kao Yang, Chi-Min Hwu, Ching-Lan Cheng

**Affiliations:** 1 Department of Ophthalmology, Kaohsiung Veterans General Hospital, Kaohsiung, Taiwan; 2 School of Medicine, National Yang-Ming University School of Medicine, Taipei, Taiwan; 3 Department of Pharmacy, College of Medicine, National Cheng Kung University Hospital, National Cheng Kung University, Tainan, Taiwan; 4 School of Pharmacy and Institute of Clinical Pharmacy and Pharmaceutical Sciences, College of Medicine, National Cheng Kung University, Tainan, Taiwan; 5 Section of Endocrinology and Metabolism, Department of Medicine, Taipei Veterans General Hospital, Taipei, Taiwan

**Keywords:** Diabetic retinopathy, Detection bias, NHIRD, Pay-for-performance

## Abstract

**Background:**

Pay-for-Performance programs have shown improvement in indicators monitoring adequacy and target achievement in diabetic care. However, less is known regarding the impact of this program on the occurrence and long-term effects of diabetic retinopathy. The objective of this study was to determine the effect of pay-for-performance program on the development of treatment needed for diabetic retinopathy in type 2 diabetes patients.

**Methods:**

We conducted a nationwide retrospective cohort study with a matching design using the Taiwan National Health Insurance Research Database from 2000 to 2012. The outcome was defined as the treatment needed diabetic retinopathy. We matched Pay-for-Performance and non-Pay-for-Performance groups for age, gender, year diabetes was diagnosed and study enrollment, and duration of follow-up.

**Results:**

A total of 9311 patients entered the study cohort, of whom 2157 were registered in the Pay-for-Performance group and 7154 matched in the non-Pay-for-Performance group. The incidence of treatment needed diabetic retinopathy was not significantly different in two groups. However, the incidence of treatment needed diabetic retinopathy was significantly different if restricted the non-Pay-for-Performance group who had at least 1 eye examination or optical coherence tomography within 1 year (adjusted hazard ratio, 0.78; 95% confidence interval, 0.64–0.94).

**Conclusions:**

Pay-for-Performance is valuable in preventing the development of treatment needed diabetic retinopathy, which could be attributed to the routine eye examination required in the Pay-for-Performance program. We could improve our diabetic care by promoting eye health education and patient awareness on the importance of regular examinations.

**Electronic supplementary material:**

The online version of this article (10.1186/s12913-018-3454-6) contains supplementary material, which is available to authorized users.

## Background

Type 2 diabetes mellitus (T2DM) is a chronic disease with multiple complications if not appropriately controlled, which mainly include macrovascular and microvascular diseases. Because the prevalence of T2DM has been increasing worldwide during the past decades, care for T2DM patients poses a critical burden on the health care system [1, 2]. Diabetic retinopathy (DR) is one of the most common complications of diabetes and is a leading cause of severe visual impairment among working-age adults worldwide [3]. Globally, it has been estimated that approximately 30% of diabetes have DR, and one-third of DR is sight-threatening diabetic retinopathy (STDR) [4]. Indeed, visual loss is associated with a substantial and measurable diminution in quality of life [5]. This diminution in quality of life can be directly compared with that induced by systemic health states [6]. A large-scale longitudinal study showed that increased health costs are associated with progressive DR in diabetic enrolees the National Health Insurance Program in Taiwan [7].

It is known that optimal glycemic control can slow the progression to diabetes-related co-morbidities [8, 9]. Evidence has also shown that the incidence of DR is related to control of glycated hemoglobin (HbA1C), blood pressure, and cholesterol. Furthermore, group visits have been reported to improve the efficacy and quality of diabetic care [10]. Hence, care systems have been developed for diabetic patients. Pay-for-performance (P4P) programs with financial incentives have been advocated to promote the quality of care through target achievement according to recommendations, thus reducing healthcare expenditures [11]. The National Health Insurance Program in Taiwan introduced a voluntary incentive program in 2001 [12]. Process-based outcomes of P4P included periodic examination of glycated hemoglobin (A1C), blood pressure, eye examinations, lipid, urinalysis, and foot examination. Service fees are reimbursed to process-based outcomes and bonuses for those who achieve better intermediate clinical indicators, such as HbA1C and low-density lipoprotein. Participating physicians and diabetes educators are required to complete specialized training in diabetes care and physicians can select patients at their discretion. P4P programs in the United Kingdom have shown improvement in indicators monitoring adequacy and target achievement, including HbA1C, cholesterol, and blood pressure [11, 13]. The risk of DM-related macrovascular complications, such as cardiovascular disease, stroke, and all-cause mortality, were also reduced in P4P enrollees [14, 15]. The inclusion of T2DM patients in the P4P program gives the opportunity for early detection of DR and treatment; however, less is known regarding the impact of the P4P program on the occurrence and long-term effects of DR. We determined whether or not a P4P program under Taiwan’s National Health Insurance Program decreased the risk of sight-threatening complications in T2DM.

## Methods

### Data source

The National Health Insurance Research Database (NHIRD) is a population-based claims database, and covers > 99.9% of population in Taiwan. The computerized de-identified databases were derived from the Bureau of National Health Insurance, Taiwan. Since 2000, the NHIRD has been maintained and entrusted to the National Health Research Institutes, and is available for research purposes. The NHIRD compiles information on the demographic information of enrollees, details of care facilities, physician specialists, and contracted pharmacies, and service claims from the outpatient department and ambulatory care. Linkage between different NHIRD datasets can be achieved by the unique and anonymous identifiers created by the NHRI. For the current study, we used the Longitudinal Health Insurance Databases (LHID2010), which is a cohort of 1 million beneficiaries randomly sampled from the 2010 registry, and retrieve all medical records from year 2000 to 2012. The details of the sampling process can be found at the Taiwan National Health Research Institute website, and there are no significant differences in the distributions of age and gender between the patients in the sampled databases and the original NHIRD [16]. Researchers using the NHIRD are required to sign a written declaration not to gather information that could violate patients’ or care providers’ privacy. At the same time, researchers should follow closely the Taiwan Personal Information Protection Act. The hospital Institutional Review Board and Ethics Committee approved this study, which adhered to the tenets of the Declaration of Helsinki; patient consent was not required (VGHKS14-CT3–04).

### Matched cohort design and study population

We performed a nationwide retrospective cohort study with a matching design using the data from 2000 to 2012. The inclusion criteria of the study subjects included those who had at least 2 diabetes (ICD-9250.xx) outpatient visits or had at least 1 diabetes (ICD-9250.xx) inpatient admission within 1 year, but never had any T1DM outpatient or inpatient visits (ICD-9250.× 1, 250.× 3) between 2002 and 2006. The first visit for diabetes was defined as the enrollment date. A new diagnosis of T2DM included patients who received anti-diabetic medications (including insulin) within 1 year after the date of enrollment and no diabetes visits 2 years before the enrollment date. Diabetic patients who met with the following criteria were eligible to enter the P4P program: 2 medical consultations for diabetes within 90 days; and 1 inpatient admission and 2 ambulatory consultations for diabetes within 1 year. Patients who met the following criteria were excluded: participated in the P4P program before the enrollment date, which might be due to an incomplete diagnosis; participated in the P4P program within 365 days after enrollment, because the first year after T2DM diagnosis was used to examine compliance; did not have any medical visit within 365 days after the enrollment date; and had a follow up duration ≤365 days after the enrollment date. We used the chronic illness with complexity index (CIC) and diabetes complication severity index (DCSI) to assessed disease severity. CIC was used to adjust complexity of comorbidity in patients with multiple chronic conditions. The score included complexity information on non-diabetes physical illness, diabetes-related and mental illness/substance abuse domain [17]. DCSI was calculated by DM complications, including domains of retinopathy, neuropathy, cerebrovascular, cardiovascular, peripheral vascular disease, and metabolic. Each complication was given a score of zero to two according to the severity and summed up to give the DCSI [18].

### Exposure definition

**Fig. 1:**
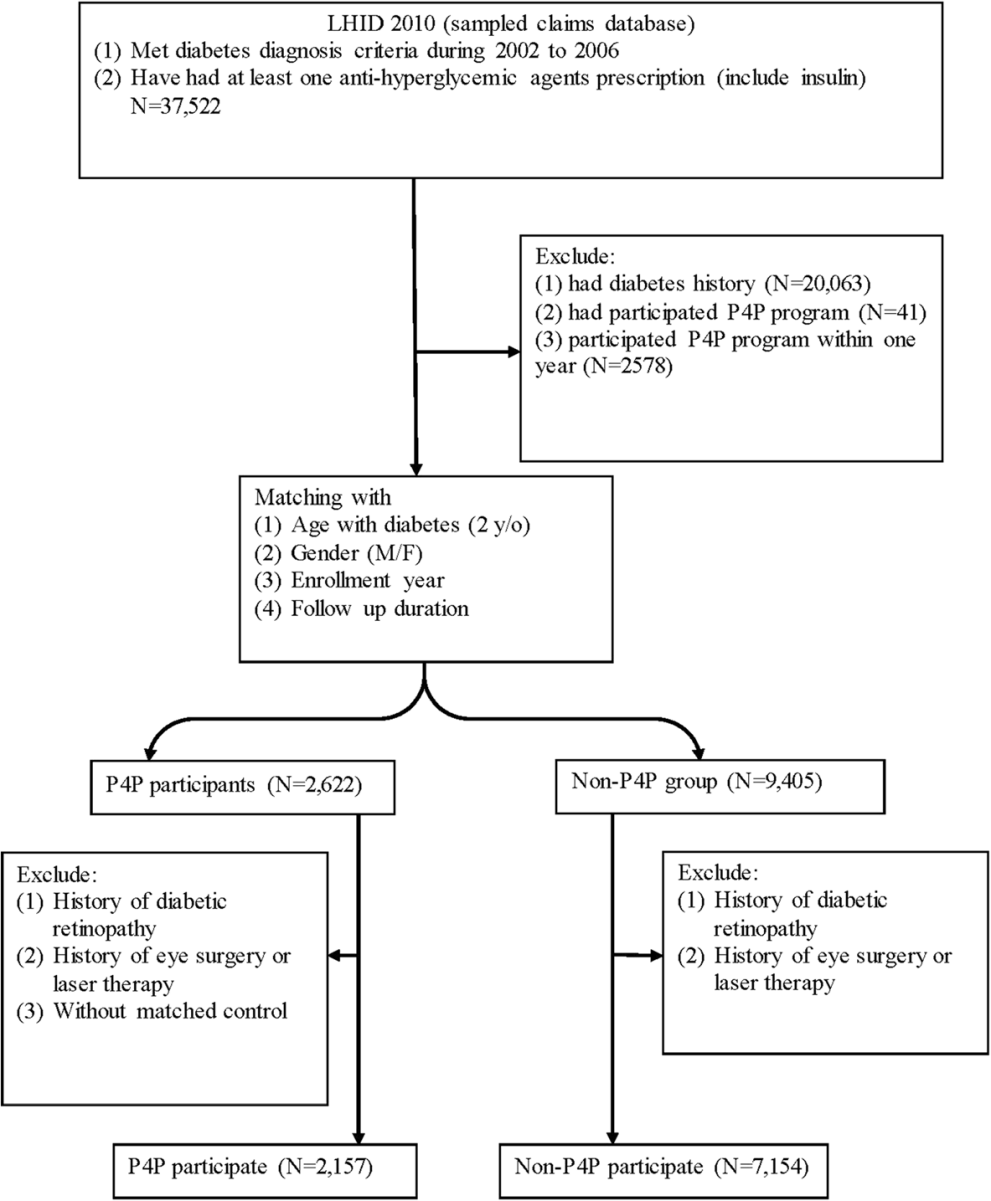
The algorithm of cohort assembly

We matched P4P and non-P4P groups (matching ratio, up to 1:3) within the enrolled age (+/− 2 years), gender (Male/Female), year of diabetes diagnosis and enrollment, and duration of follow up. The index date for the P4P group was the first date the P4P program was entered as recorded for reimbursement, and the index date was used to match subjects for the non-P4P groups. This means the index date was the same date in P4P and non-P4P group for each matched pairs. After the assigned index date for each subject, we excluded patients who had a history of DR, eye surgery or a laser procedure within 2 years before the index date, and patients in the P4P group who did not have any non-P4P matches. There were 2157 patients in the P4P group and 7154 patients in the non-P4P group enrolled in this cohort study (Fig. 1).

### Outcome definition

The outcome was defined as incidence of treatment needed diabetic retinopathy (TNDR), thus patients who had clinic visits with severe DR (ICD-9362.02 [proliferative diabetic retinopathy] or 362.07 [diabetic macular edema]) and received surgery or laser therapy within 90 days. We followed patients from the index date until the outcome, death, or end of the study period (December 2012), whichever came first. In order to avoid overestimating the incidence of DR by retrieving diagnostic codes alone, we investigated the effect of P4P on the development of TNDR by combining the diagnosis code and procedure code to avoid the outcome bias.

### Statistical method

A paired Student’s *t-*test and McNemar’s test were used to compare the means and proportions of baseline characteristics. The Kaplan-Meier method and log-rank test were used to estimate the event-free survival rate and to examine differences in the risk of TNDR between the P4P and non-P4P groups. Crude incidence rates were calculated as the observed number of patients with TNDR divided by 1000 person-years of follow-up. Cox proportional regression models with stepwise selection were used for adjusted time-to-event analyses of the outcome. The model selection criteria at a significance level of 0.3 was required to allow a variable into the model, and a significance level of 0.35 was required for a variable to remain in the model. Cox proportional hazards assumption assessment used cumulative sums of martingale residuals, and the Kolmogorov-type supremum test was based on a sample of 1000 simulated residual patterns. Time-dependent Cox regression models were used to estimate adjusted hazard ratios (aHRs) if the proportional hazard assumption was violated. The hazard ratio (HR) and 95% confidence interval (CI) were calculated by the matching method using the SAS PHREG procedure with a strata statement to evaluate the association between the P4P program and occurrence of TNDR. A *P* value ≤0.05 was considered statistically significant.

We performed several sensitivity analyses to determine the robustness of our findings. First, because compliance may affect diabetic control, we evaluated the baseline compliance of diabetes medications 1 year prior to the index date using the medication possession ratio (MPR). In literature, MPR ≥ 80% has been proposed as good medication adherence for chronic disease such as diabetes mellitus and hypertension [19, 20]. Because the medication refill in Taiwan is a 3-month supply, we defined the MPR to be the ratio of total days of DM medication supply during one-year period. We then selected patients with a MPR ≥ 80% as good compliance to avoid selection bias. Second, we determined the propensity score (PS) with a 1:1 matching method to balance the characteristics between the P4P and non-P4P groups. We used multivariable logistic regression models to estimate propensity score (PS), and all baseline characteristics (listed at Table 1) as covariates included in the model. Patients were matched by PS using a greedy 5-to 1-digit matching algorithm. Third, regular eye examinations were required to comply with the P4P program, thus we restricted the cohort population that had fundus examinations or optical coherence tomography (OCT) scans within 1 year after the index date, which was the requirement for eligible P4P patients at the initial visit, to avoid detection bias. These statistical analyses were performed using SAS 9.4 software (SAS Institute Inc., Cary, NC, USA).

**Table 1:** Baseline characteristics of the matched cohort patients with type 2 diabetes

Characteristics	P4P		Non-P4P		P*
	*N* = 2157		*N* = 7154		
Diagnosed DM Age (years)	54.30±12.9		54.09±12.4		< 0.0001
Gender (Female)	977	(45.3)	3222	(45.0)	–
Enrollment year					–
2002	462	(21.4)	1402	(19.6)	
2003	477	(22.1)	1526	(22.1)	
2004	441	(20.4)	1495	(20.9)	
2005	404	(18.7)	1397	(19.5)	
2006	373	(17.3)	1334	(18.6)	
Concomitant diseases (previous one year)					
Admission for CHF	243	(11.3)	795	(11.1)	0.8644
Admission for Stroke	53	(2.5)	286	(4.0)	0.001
Chronic kidney disease	146	(6.8)	439	(6.8)	0.2853
DM neuropathy	278	(12.9)	485	(6.8)	< 0.0001
DM nephropathy	232	(10.7)	369	(5.1)	< 0.0001
Liver disease	597	(27.7)	1474	(20.6)	< 0.0001
Hypertension	1318	(61.1)	4096	(57.2)	0.0002
CIC score					0.2760
Mean (range)	0.20	(0–5)	0.18	(0–8)	
0	1897	(87.9)	6397	(89.4)	
1	139	(6.4)	412	(5.7)	
2	76	(3.5)	222	(3.1)	
> 2	45	(2.1)	123	(1.7)	
DCSI score					< 0.0001
Mean (Range)	0.79	(0–10)	0.65	(0–11)	
0	1215	(56.3)	4737	(66.2)	
1	524	(24.3)	1184	(16.5)	
2	226	(10.5)	692	(9.7)	
> 2	192	(8.9)	541	(7.6)	
Concomitant medication (previous 180 days)					
ACEI/ARB	914	(42.4)	2385	(33.3)	< 0.0001
Anti-coagulant agents	20	(0.93)	81	(1.1)	0.3978
Alpha-antagonists	83	(3.8)	240	(3.3)	0.2478
Beta-blocking agents	502	(23.3)	1681	(23.5)	0.9560
CCB	661	(30.6)	2098	(29.3)	0.1348
Diuretics	381	(17.6)	981	(13.7)	< 0.0001
Digoxin	32	(1.5)	97	(1.4)	0.5967
Insulin	247	(11.5)	210	(2.9)	< 0.0001
Lipid lowering agents	923	(42.8)	1903	(26.6)	< 0.0001
Metformin	1719	(79.7)	3422	(47.8)	< 0.0001
Sulfonylurea	1591	(73.7)	3683	(51.5)	< 0.0001
Systemic corticosteroids	348	(16.1)	1057	(14.8)	0.1541

Data are presented as mean (S.D.) or *n* (%). *P4P* Pay-for-performance program, *CIC* Chronic illness with complexity index, *DCSI* Diabetes complication severity index

*matched paired-test

## Results

We identified 17,418 newly diagnosed patients with T2DM in the LHID2010 between 2002 and 2006. A total of 9311 patients entered the study cohort, of whom 2157 were registered in the P4P program and 7154 were matched in the non-P4P groups (Fig. 1). The baseline characteristics of the patients are shown in Table 1. The mean age was 54.30±12.9 years in the P4P group and 54.09±12.4 years in the non-P4P group. Males were dominant in our cohort population. As compared with the patients in the non-P4P group, the P4P patients had more liver disease (*p* < 0.0001), hypertension (*p* = 0.0002), and diabetes related complications, such as diabetic neuropathy (*p* < 0.0001) and diabetic nephropathy (*p* < 0.0001). The P4P group used more concomitant medications including diuretics (*p* < 0.0001), insulin (*p* < 0.0001), lipid-lowering agents (*p* < 0.0001), metformin (p < 0.0001), and sulfonylureas (*p* < 0.0001), compared with the non-P4P group. We assessed disease severity using CIC and DCSI, and found that the P4P group had more severe diabetes complications (*p* < 0.0001) than the non-P4P group.

**Fig. 2:**
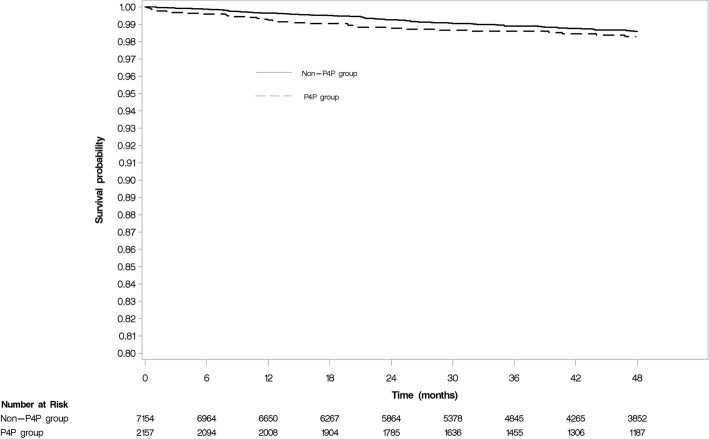
The survival analysis of DR risk

The effects of the P4P program on outcomes are shown in Table 2 and Fig. 2. We observed that 43 events occurred in the P4P group (4.53 per 1000 patient-years) and 119 events occurred in the non-P4P group (3.84 per 1000 patient-years) during the follow-up period. The incidence of TNDR was not significantly different between the P4P and non-P4P group (aHR, 0.99; 95% CI, 0.93–1.05).

**Table 2:** Incidence of Treatment Needed Diabetic Retinopathy (TNDR) and sensitivity analysis, data presented as patient number (incidence rate/1000 person-year)

	P4P group	Non-P4P group	Unadjusted HR (95% CI)	Adjusted HR^*^ (95% CI)
TNDR	*N* = 2157 Event = 43 (4.53/1000 person-year)	*N* = 7154 Event = 119 (3.84/1000 person-year)	1.17 (0.82–1.68)	0.99 (0.93–1.05)^a^
Sensitivity analysis				
Prior MPR > 80%	*N* = 1622 Event = 26 (3.67/1000 person-year)	*N* = 3871 Event = 60 (3.67/1000 person-year)	0.98 (0.58–1.63)	0.95 (0.87–1.05)^b^
Propensity score matching	N = 1790 Event = 39 (4.88/1000 person-year)	*N* = 1790 Event = 67 (8.64/1000 person-year)	–	0.63 (0.39–1.00)
Restrict eye exam within one year in Non-P4P group	*N* = 2157	*N* = 734	0.44 (0.28–0.71)	0.78 (0.64–0.94)^c^
	Event = 43 (4.53/1000 person-year)	Event = 31 (10.29/1000 person-year)		

*P4P as time-dependent variable

^a^Adjusted variables: age, insulin, sulfonylurea, metformin, liver disease, CIC score and DCSI score

^b^Adjusted variables: age, corticosteroid, insulin, sulfonylurea, metformin, alpha-blockers, CIC score, DCSI score, history of admission for stroke and heart failure, DM nephropathy

^c^Adjusted variables: age, insulin, sulfonylurea, metformin, beta-blockers, CIC score, DCSI score, liver disease, renal disease, admission for heart failure

Table 2 shows the sensitivity results. The rates of good adherence (MPR > 80%) among the P4P group (1622/2157 = 75%) was higher than the non-P4P group (3871/7154 = 54%); however, restriction to good adherence patients reduced selection bias and did not change the estimates of the P4P program. (aHR, 0.95; 95% CI, 0.80–1.05). The prevalence of co-morbidities and co-medications did not differ between the P4P and non-P4P groups after PS matching (Additional file 1: Table S1). In the PS model, point estimates were consistently lower in the P4P groups, though confidence intervals were cross 1 means. The differences were not significant (aHR, 0.64; 95% CI, 0.39–1.00); however, there was a trend toward favoring the P4P group. Restriction of the non-P4P group participants who had at least 1 eye examination yielded 734 (10.2%) patients. The crude and aHR for the incidence of TNDR was significantly lower in the P4P group compared with the non-P4P groups (aHR, 0.78; 95% CI, 0.64–0.94). We further checked the detection bias in eye examinations versus no eye examinations among the non-P4P group. Non-P4P patients who had eye examinations had a higher rate of TNDR compared with those having no eye examination (aHR, 1.24; 95% CI, 1.08–1.42; Additional file 2: Table S2).

## Discussion

Our study results showed significant differences on the incidence of TNDR after we restricted the non-P4P patients who that had at least 1 eye examination or OCT within 1 year. This imply the detection bias should be considered in observational study. Detection bias can occur in studies when collection outcome information in groups is differ, such as screening examination for cancer detection. The association between pioglitazone and bladder cancer is one of example. The frequency of physician visits is more in pioglitazone users than comparison groups, lead to the discovery undiagnosed bladder cancer and result in a misleading risk of pioglitazone. [21] There were only 734 patients (10.2%) in the non-P4P group who met this requirement (at least 1 eye examination or OCT within 1 year) and were included in the subgroup analysis. Due to the low number of patients included, we did not conduct PS matching in the subgroup analysis. The difference between the P4P and non-P4P groups was expected to be less if more patients in the non-P4P group had a received eye examination before symptoms developed and were included in the subgroup analysis because we used TNDR as an outcome parameter.

A P4P program in the health care system is one of many quality improvement strategies, which provide financial rewards to encourage proven high-quality care to patients, especially patients with chronic diseases [14, 15]. The effect of this program for diabetes care is not conclusive due to various study designs and outcome evaluations amongst the published studies. Oluwatowoju, et al. conducted a retrospective cohort study among patients who completed 2 years of follow-up to evaluate the effect of Quality Outcome Framework (QOF) in diabetic care. The proportion of subjects achieving the target quality indicator (HbA1c < 7.5% and total cholesterol < 5.0 mmol/l) increased after 2 years of follow-up [11]. Another study evaluated the effect of QOF in diabetic care using a quasi-experimental design with an interrupted time series analysis. The findings did not support the notation that QOF has an effect on the HbA1c level [13]. Our outcome, TNDR (defined by diagnosis and procedure codes) should be less than the actual number of STDR, but more specific for the severity of DR in a large database. The enrollees in the P4P group were required to receive regular eye examinations. The incidence of DR was expected to be high because the diagnosis was documented for the reimbursement of the examination. In contrast, only 10.2% of patients in the non-P4P group received eye examinations during our study period. Most of these patients visit ophthalmologists only when ocular symptoms develop, and tend to be treatment needed. If all of the patients in the non-P4P group had routine eye examinations before the symptoms developed, the incidence of TNDR should have been much lower than the present data showed. Our results revealed the fact that routine eye examinations might be as effective as P4P in preventing TNDR.

An important issue in using the P4P program in the DR study is the choice of adequate comparable control groups. Because the P4P program is a volunteer incentive plan, patients with good compliance or disease awareness might be more willing to be enrolled. Two criteria have to be met to participate in the P4P program: 1) patients had to visit the same physician at least twice within 90 days and have the diagnosis of diabetes mellitus at visits; and 2) at least one diabetes admission in the hospital and at least 2 medical visits within 1 year at the same hospital. Patient enrollments are determined by the physician. One study reported that patients enrolled in the Taiwan P4P program had fewer co-morbidities than non-enrolled patients, which suggests that providers had discretion in choosing patients for this program [22]. Other study showed the physicians participated in P4P program were younger, more in endocrinology specialty, public, urban and regional hospitals [23]. Another study showed that patient self-care and the adherence rate were better in participants in the P4P program for at least 1 year than new participants (≤ 3 month), which suggests that the effect of the P4P program is time-dependent [24]. Thus, we conducted this matched cohort for at least 1 year observational period to have an equal chance to join the P4P program, and assigned the index date for the non-P4P group with the same baseline T2DM diagnosis period.

It is known that the critical factors required for success of a DR screening program is patient adherence to recommendations for follow-up care. Cost and accessibility have been cited as major barriers to eye care adherence by diabetic patients in surveys [25]. However, the DR screening program in a public clinic largely serving an African American population showed low adherence to interval recommendations for follow-up eye appointments was an important factor, even though low cost and high accessibility [26]. Under the P4P and National Health Insurance system in Taiwan, the eye exam are usually arranged by the physician within the same hospital and the cost was all covered the insurance. If patients chose to have the eye exam at local clinic, they only have to pay the register fee, which was only about 3–5 USD. We think that the barrier of cost and accessibility to the eye exam was very low in Taiwan. Our results echo the conclusion that incorporation of eye health education initiatives are important in promoting adherence to recommended comprehensive eye care for preventing vision loss. The P4P program needs individual patient attendance as well as a group of high-cost staff, including physicians, certified health educators, social workers, and licensed dieticians. However, patients might miss the scheduled appointment due to the transportation expense and the patients’ wage might be reduced if he/she attended the appointment and was absent from work. Patients might misleading the level of care needed and loss some appointments resulted in diabetic complication. To compensate for this difficulty, another option may be e-learning through innovative applications available through smart-technologies that can be integrated into a patient’s day to help increase adherence. This concept is still very novel, but with advances in technology and decreasing costs in production this may be an option in the future to help individuals with their health. For a highly-motivated patient, a reminder telephone call or message through a social network app to visit a nearby ophthalmologist might be just as effect.

Our study is the first to evaluate the association between T2DM P4P and risk of diabetic related retinopathy by TNDR, but not DR diagnosis code only. Taiwan’s national health insurance covered nearly all of the medical costs, all of the medical services for treatment would be recorded and detected based on our method. Studying patients in the NHI database allows for the easy collection of a large number of cases and long-term observation. As shown by our data, the incidence of TNDR was less than STDR reported in the literature, but more specific for the severity of DR in a large database. The incidence of DR in the studies based on diagnosis code only might be overstated due to the reimbursement system. Our study truly reflected the status of severe diabetic retinopathy in Taiwan. Regular eye examination is the essential practice to detect diabetic retinopathy and required in the P4P program in Taiwan NHI. In addition, patient consultation delivered in the P4P program would also reinforces modification of life style and medication compliance, and thus is valuable in preventing the development of TNDR.

Our study had limitations. First, DR was not validated in most of the insurance database studies. Because the diagnosis code is related to the reimbursement, diagnosis is sometimes overstated. Hence, we determined the effect of P4P and medication adherence on the development of TNDR by combining the diagnosis and procedure codes to avoid outcome bias. By using this protocol, we might miss some mild DR, but all treatment-needed events should have been identified in this study. Because some types of DR (mild and moderate nonproliferative diabetic retinopathy) are generally not treated, treatment-needed reflects the severity and need of intervention [27]. Meanwhile, we defined the DR cases that needed treatment by using laser or surgery within 90 days after DR diagnosis, which truly reflected the group with severity. Second, there were some unmeasured confounding factors we were not able to adjust. DM severity was unknown due to a lack of laboratory data, such as HbA1c and, renal function in the NHIRD. These factors were documented as risk for the development and progression of DR. The personal factors (smoking, alcohol consumption, or economic status) or medication been actually taken, which might affect the risk of DR, were not known. Although we performed sensitivity analysis by using PS matching to balance the characteristics, the unmeasured confounding factors might still bias the results. Finally, we enrolled patients with T2DM on medications. The effect of the P4P program on T1DM cases and lifestyle-controlled DM cases was not investigated.

## Conclusions

Pay-for-Performance is valuable in preventing the development of treatment needed diabetic retinopathy, which could be attributed to the routine eye examination required in the Pay-for-Performance program. We could improve our diabetic care by promoting eye health education and patient awareness on the importance of regular examinations.

## Additional files

Additional file 1: **Table S1.** Baseline characteristics of the cohort patients with type 2 diabetes using propensity score (DOCX 16 kb)

Additional file 2: **Table S2.** Incidence of diabetic retinopathy in eye examination groups compared with no eye examination groups among non-P4P population (check detection bias) (DOCX 15 kb)

## Abbreviations

aHRs: Adjusted hazard ratios
CI: Confidence interval
CIC: Chronic illness with complexity
DCSI: Diabetes complication severity index
DM: Diabetes mellitus
DR: Diabetic retinopathy
HbA1C: Glycated hemoglobin
HRs: Hazard ratios
LHID: Longitudinal Health Insurance Databases
MPR: Medication possession ratio
NHIRD: National Health Insurance Research Database
OCT: Optical coherence tomography
P4P: Pay-for-Performance
PS: Propensity score
QOF: Quality Outcome Framework
STDR: Sight-threatening diabetic retinopathy
T2DM: Type 2 diabetes mellitus
TNDR: Treatment needed diabetic retinopathy
