# Supplementary material for: Pay for performance program reduces treatment needed diabetic retinopathy - a nationwide matched cohort study in Taiwan
Source: BMC Health Serv Res. 2018 Aug 15;18:638. doi: 10.1186/s12913-018-3454-6 (PMC6094472; doi:10.1186/s12913-018-3454-6)
Supplement: Supplementary file 1 — Table S1. Baseline characteristics of the cohort patients with type 2 diabetes using propensity score (DOCX 16 kb) [file 12913_2018_3454_MOESM1_ESM.docx]

Additional file 1: **Table S1** Baseline characteristics of the cohort patients with type 2 diabetes using propensity score

|  |  | | | | |  |
| --- | --- | --- | --- | --- | --- | --- |
| Characteristics | P4P  N=1,790 | | | Non-P4P  N=1,790 | | P |
| **Diagnosed DM Age (years)** | | 55.05±12.7 | | 55.35±11.7 | | 0.4604 |
| **Gender (Female)** | | 961 | (53.7) | 983 | (54.9) | 0.460 |
| **Enrollment year** | |  |  |  |  | - |
| 2002 | | 462 | (21.4) | 1402 | (19.6) |  |
| 2003 | | 477 | (22.1) | 1526 | (22.1) |  |
| 2004 | | 441 | (20.4) | 1495 | (20.9) |  |
| 2005 | | 404 | (18.7) | 1397 | (19.5) |  |
| 2006 | | 373 | (17.3) | 1334 | (18.6) |  |
| **Concomitant diseases (previous one year)** | | | | | | |
| Admission for CHF | | 213 | (11.9) | 205 | (11.5) | 0.6772 |
| Admission for Stroke | | 47 | (2.6) | 44 | (2.5) | 0.7501 |
| Chronic kidney disease | | 122 | (6.8) | 110 | (6.1) | 0.4153 |
| DM neuropathy | | 200 | (11.2) | 187 | (10.5) | 0.4841 |
| DM nephropathy | | 170 | (9.5) | 146 | (8.2) | 0.1574 |
| Liver disease | | 462 | (25.8) | 456 | (25.5) | 0.8184 |
| Hypertension | | 1105 | (61.7) | 1121 | (62.6) | 0.5813 |
| **CIC score** | |  |  |  |  | 0.8465 |
| Mean (range) | | 0.19 | (0-3) | 0.18 | (0-3) |  |
| 0 | | 1583 | (88.4) | 1587 | (88.6) |  |
| 1 | | 106 | (5.9) | 111 | (6.2) |  |
| 2 | | 63 | (3.5) | 54 | (3.0) |  |
| >2 | | 38 | (2.1) | 38 | (2.1) |  |
| **DCSI score** | |  |  |  |  | 0.4656 |
| Mean (Range) | | 0.71 | (0-3) | 0.68 | (0-3) |  |
| 0 | | 1017 | (56.8) | 1057 | (59.0) |  |
| 1 | | 423 | (23.6) | 389 | (21.7) |  |
| 2 | | 187 | (10.5) | 191 | (10.6) |  |
| >2 | | 163 | (9.1) | 153 | (8.5) |  |
| **Concomitant medication (previous 180 days)** | | | | | | |
| ACEI/ARB | | 764 | (42.7) | 757 | (42.2) | 0.8129 |
| Anti-coagulant agents | | 20 | (1.1) | 25 | (1.4) | 0.4532 |
| Alpha-antagonists | | 67 | (3.7) | 66 | (3.7) | 0.9296 |
| Beta-blocking agents | | 432 | (24.1) | 478 | (26.7) | 0.0774 |
| CCB | | 562 | (31.4) | 576 | (32.2) | 0.6153 |
| Diuretics | | 314 | (17.5) | 303 | (16.9) | 0.6264 |
| Digoxin | | 25 | (1.4) | 28 | (1.6) | 0.6780 |
| Insulin | | 133 | (7.4) | 117 | (6.5) | 0.2941 |
| Lipid lowering agents | | 737 | (41.2) | 708 | (39.6) | 0.3232 |
| Metformin | | 1,397 | (78.0) | 1,427 | (79.7) | 0.2193 |
| Sulfonylurea | | 1,292 | (72.2) | 1,331 | (74.4) | 0.1408 |
| Systemic corticosteroids | | 296 | (16.5) | 295 | (16.5) | 0.9641 |

Data are presented as mean (S.D.) or n (%). P4P, pay-for-performance program. CIC, chronic illness with complexity index. DCSI, diabetes complication severity index.
