# Supplementary material for: Pay for performance program reduces treatment needed diabetic retinopathy - a nationwide matched cohort study in Taiwan
Source: BMC Health Serv Res. 2018 Aug 15;18:638. doi: 10.1186/s12913-018-3454-6 (PMC6094472; doi:10.1186/s12913-018-3454-6)
Supplement: Supplementary file 2 — Table S2. Incidence of diabetic retinopathy in eye examination groups compared with no eye examination groups among non-P4P population (check detection bias) (DOCX 15 kb) [file 12913_2018_3454_MOESM2_ESM.docx]

Additional file 2: **Table S2** Incidence of diabetic retinopathy in eye examination groups compared with no eye examination groups among non-P4P population (check detection bias)

|  | No eye examination  N=6,420 | Have eye examination  N=734 | Unadjusted HR  (95% CI) | Adjusted HR*  (95% CI) |
| --- | --- | --- | --- | --- |
| DR event | Event=88  (95/1000 person-year) | Event=31  (313/1000 person-year) | 6.50 (3.03-13.94) | 1.24 (1.08-1.42) |

Eye examination: indirect ophthalmoscopy (23702C)、funduscopic exam (23501C)、fundus color photo picture (23502C)

*adjusted variables: age at first DM diagnosis, gender, DM-nephropathy, DM-neuropathy, insulin use, CIC score and DCSI score; eye examination as time-dependent variable
